# Dietary supplements and vascular function in hypertensive disorders of pregnancy

**DOI:** 10.1007/s00424-023-02810-2

**Published:** 2023-04-12

**Authors:** Andy W C Man, Yawen Zhou, Ning Xia, Huige Li

**Affiliations:** Department of Pharmacology, Johannes Gutenberg University Medical Center, Langenbeckstr. 1, 55131 Mainz, Germany

**Keywords:** Nitric oxide, Endothelium, Fetal reprogramming, Cardiovascular diseases, Placenta

## Abstract

Hypertensive disorders of pregnancy are complications that can lead to maternal and infant mortality and morbidity. Hypertensive disorders of pregnancy are generally defined as hypertension and may be accompanied by other end organ damages including proteinuria, maternal organ disturbances including renal insufficiency, neurological complications, thrombocytopenia, impaired liver function, or uteroplacental dysfunction such as fetal growth restriction and stillbirth. Although the causes of these hypertensive disorders of pregnancy are multifactorial and elusive, they seem to share some common vascular-related mechanisms, including diseased spiral arteries, placental ischemia, and endothelial dysfunction. Recently, preeclampsia is being considered as a vascular disorder. Unfortunately, due to the complex etiology of preeclampsia and safety concerns on drug usage during pregnancy, there is still no effective pharmacological treatments available for preeclampsia yet. An emerging area of interest in this research field is the potential beneficial effects of dietary intervention on reducing the risk of preeclampsia. Recent studies have been focused on the association between deficiencies or excesses of some nutrients and complications during pregnancy, fetal growth and development, and later risk of cardiovascular and metabolic diseases in the offspring. In this review, we discuss the involvement of placental vascular dysfunction in preeclampsia. We summarize the current understanding of the association between abnormal placentation and preeclampsia in a vascular perspective. Finally, we evaluate several studied dietary supplementations to prevent and reduce the risk of preeclampsia, targeting placental vascular development and function, leading to improved pregnancy and postnatal outcomes.

## Introduction

According to the World Health Organization (WHO), the worldwide maternal mortality is unacceptably high [1]. In 2017, approximately 810 women died each day from pregnancy-related complications [1]. Maternal mortality is the result of complications during and following pregnancy and childbirth. Most of these complications develop during pregnancy while some complications may exist before pregnancy and are worsened during pregnancy. The major complications that lead to most maternal deaths include severe bleeding during childbirth, infections, unsafe abortion, high blood pressure during pregnancy, and other causes associated with chronic diseases like diabetes [2]. Indeed, most of these complications are largely preventable and can be improved with better hygiene condition and healthcare facilities. However, among these complications, hypertensive disorders of pregnancy, including gestational hypertension, pre-eclampsia, and eclampsia, can only be minimized via pharmacological and dietary interventions. Also, pregnancy-associated hypertensive disorders occur in approximately 10% of all pregnancies and account for approximately 14% of maternal mortality [3, 4]. Hypertensive disorders of pregnancy are generally defined as hypertension (≥ 140 mmHg systolic and/or ≥ 90 mmHg diastolic blood pressure) occurring after 20 weeks of gestation while preeclampsia is defined as gestational hypertension accompanied by proteinuria (excretion of ≥ 300 mg protein every 24 h) [5]. Apart from maternal mortality, these hypertensive disorders of pregnancy can lead to an increased risk for future metabolic and cardiovascular disease for both mother and offspring [6, 7]. Therefore, the identification of better preventive strategies and treatment modalities is imperative to minimize these inter-generational pathological consequences of pregnancy-related complications. Despite the fact that the causes of these hypertensive disorders of pregnancy are multifactorial and elusive, they seem to share some common vascular-related mechanisms, including diseased spiral arteries, placental ischemia, and endothelial dysfunction [8–10]. Various studies have shown that dietary patterns and supplements can influence adverse pregnancy and birth outcomes [11–13]. This review aims to summarize the current understanding of the beneficial effects of different dietary supplementations in hypertensive disorders of pregnancy, focusing on vascular function.

## Hypertensive disorders of pregnancy and vascular function

The exact mechanisms underlying the development of hypertensive disorders of pregnancy are unclear. Currently, most of the literature has specifically focused on the pathogenesis underlying preeclampsia, while few of them have discussed on gestational hypertension, although gestational hypertension is also associated with adverse maternal and perinatal outcomes, and similar renal histopathology [14–16]. Traditionally, significant proteinuria has been the second criterion required to distinguish between gestational hypertension and preeclampsia. Indeed, various maternal and fetal consequences now appear in international guidelines for the diagnosis of preeclampsia, including hypertension and end-organ dysfunction, while proteinuria is no longer mandatory [17–19]. Therefore, the review will primarily focus on the pathogenesis of preeclampsia.

**Fig. 1:**
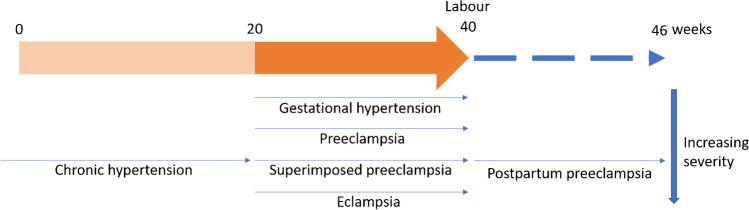
Diagram showing different hypertensive disorders of pregnancy. Gestation hypertension is the increase in blood pressure during pregnancy. Preeclampsia patients have gestation hypertension and accompanied by the occurrence of at least one of the following complications at or after 20 weeks of gestation: proteinuria, maternal organ disturbances including renal insufficiency, neurological complications, thrombocytopenia, impaired liver function, or uteroplacental dysfunction such as fetal growth restriction and stillbirth. Women with chronic hypertension who develop symptoms of preeclampsia are diagnosed with superimposed preeclampsia. Eclampsia is the development of seizures in pre-existing preeclampsia and is a life-threatening emergency condition. Postpartum preeclampsia may also occur when the symptoms of preeclampsia persist in postpartum, which may increase the risk of chronic cardiovascular diseases.

Preeclampsia is a complex and multifactorial disease defined as a rise in systolic blood pressure above 140 mmHg and/or rise in diastolic blood pressure above 90 mmHg occurring after 20 weeks of gestation in a pregnant woman without prior hypertension [5, 20], accompanied by the occurrence of at least one of the following complications at or after 20 weeks of gestation: proteinuria, maternal organ disturbances including renal insufficiency, neurological complications, thrombocytopenia, impaired liver function, or uteroplacental dysfunction such as fetal growth restriction and stillbirth [17–19]. Superimposed preeclampsia is defined as the development of any of the above-mentioned maternal organ dysfunctions in a mother with pre-existing chronic hypertension [21]. If untreated, preeclampsia can lead to eclampsia. Eclampsia is the development of seizures in pre-existing preeclampsia and is a life-threatening emergency condition that can also occur in the postpartum period [22] (Fig. 1). Although eclampsia is one of the leading causes of maternal mortality, it is not known how hypertension in pregnancy affects the cerebral circulation and causes eclampsia due to the lack of animal models [23]. The primary explanation for the pathogenesis of eclampsia is thought to be hypertensive encephalopathy [24]. In addition, preeclampsia may persist during antepartum and postpartum. Late postpartum preeclampsia is defined as the presence of preeclampsia symptoms up to 6 weeks postpartum [25]. Although the detailed pathophysiology is relatively unknown, the persistently high levels of anti-angiogenic factors after delivery may play a role in the development of postpartum preeclampsia [26]. Postpartum preeclampsia may cause endothelial damage associated with substantial maternal mortality and increased risk of chronic hypertension [25, 27].

Women with history of preeclampsia have approximately 2-fold increased risk of developing cardiovascular diseases and around 10-fold increased risk of chronic kidney diseases [28]. Also, a few epidemiologic studies have linked 15–20% of all fetal growth restriction and small for gestational age infants to preeclampsia, while 20% of all preterm births are also associated with preeclampsia [29]. Apart from obstetric and neonatal consequences, preeclampsia has also been shown to exert long-term risk of metabolic and cardiovascular complications [30]. Therefore, hypertensive disorders of pregnancy can leave long-term metabolic and cardiovascular risks to both mother and child.

Placenta plays an important role in the development and severity of preeclampsia. It has been suggested that the placenta itself, but not the fetus, is necessary for the development of preeclampsia, while preeclampsia can also occur in patients with hydatidiform moles and the removal of placenta can resolve the syndrome [31, 32]. Accumulating studies have evidenced the involvement of multifactorial mechanisms including early disturbances in placentation followed by the imbalance in angiogenic factors, aberrant inflammatory response, increased placental oxidative stress, and placental aging in the pathogenesis of preeclampsia [33, 34]. Indeed, preeclampsia can be considered as a vascular disorder. A functional and adequately vascularized placenta is crucial for healthy pregnancy and birth outcome [35]. It is proposed that the initiating step in the pathogenesis of preeclampsia is the abnormal placentation that characterized by defective trophoblast cell invasion and uterine vasculature remodeling [36]. During normal pregnancy, maternal uterine spiral arteries are remodeled into low-resistance vessels as a result of fetal trophoblast invasion and replacing the endothelial and smooth muscle cells in the vessel wall [37]. The interaction between the trophoblast and uterine natural killer cells initials the spiral artery remodeling [38]. In preeclampsia, overreaction of the maternal immunity can limit the placental development [39], while the incomplete trophoblastic invasion and spiral arteries remodeling lead to decreased placental perfusion and poor placentation [40–42], which result in the activation of pathways leading to maternal vasoconstriction and endothelial dysfunction [42]. Currently, the exact pathological mechanisms of incomplete trophoblastic invasion are not completely known while some studies have suggested, apart from immune maladaptation, the contribution of imbalanced angiogenic growth factors and placental endothelial dysfunction [40, 43].

Reduction of placental angiogenic factors plays an important role in the pathogenesis of preeclampsia. The releases of angiogenic cytokines including, vascular endothelial growth factor (VEGF), placental growth factor (PlGF), and angiopoietin 2, are required for normal placentation [40, 43, 44]. In the first trimester of pregnancy, low serum level of PlGF is detected in suspected preeclampsia [45]. Inflammation and tissue hypoxia in the spiral arteries lead to the production of the transcription factor hypoxia-inducible factor-1 (HIF-1), which in turn downregulates PlGF [46]. Also, soluble fms-like tyrosine kinase 1 (sFlt1), an antiangiogenic protein that inactivates VEGF and PlGF is upregulated in preeclampsia [47]. Therefore, placental abnormality results in decreased levels of angiogenic VEGF and PlGF and increased levels of deleterious placental factors including sFIt-1 in the maternal circulation. Moreover, the imbalance of pro- and anti-inflammatory and pro- and anti-angiogenic factors significantly contribute to generalized endothelial dysfunction, intravascular inflammation, and activation of the hemostatic system that result in the maternal syndrome [48, 49].

Throughout the whole body, the endothelium is believed to be the primary target of mediators generated from the placenta, causing endothelial dysfunction and end-organ damages [50]. Hypertension and proteinuria are common manifestation of endothelial dysfunction-mediated end-organ damages, while some severe cases of preeclampsia have microangiopathic hemolytic anemia and organ hypoperfusion [51]. Indeed, endothelial dysfunction can occur in both maternal and placental circulations in hypertension disorders of pregnancy [40–42]. In clinical studies, vascular responsiveness can be assessed by in vivo [52–55] and in vitro [56, 57] methods to examine the vascular function in normal pregnancy and preeclampsia. A clinical study has demonstrated that vascular reactivity to endothelium-dependent vasodilators (such as acetylcholine) was impaired in preeclampsia as evidenced by the reduced forearm blood flow measured by venous occlusion plethysmography in vivo [52]. Flow-mediated dilatation was reduced in preeclampsia patients compared to normal pregnant women as measured by myography in vitro [56]. In addition, a reduction of flow-mediated dilation was observed in the uterine artery or preeclampsia patients by doppler ultrasonography in vivo [54]. Also, preeclampsia is associated with a failure of shear stress-induced vasodilatation and an enhanced myogenic response in vitro, which increased the vascular resistance in the uterine circulation [57]. Serum levels of endothelial activation markers including thrombomodulin, von Willebrand factor, fibronectin, and cell adhesion molecules including intercellular adhesion molecule 1 (ICAM-1), and vascular adhesion molecule (VCAM-1) have been shown upregulated in preeclamptic patients [58–60]. Coherent with the clinical studies, treatment of recombinant sFlt-1 in human umbilical vascular endothelial cells (HUVEC) significantly increases these endothelial activation markers in vitro [61], suggesting the pathological role of sFlt-1 in endothelial activation, which in turn, contributes to the adverse responsiveness of the vasculature. During preeclampsia, patients have increased serum levels of vasoconstrictors such as endothelin 1 (ET-1) and thromboxane [62–64], while the responsiveness to vasodilators including nitric oxide (NO) and prostacyclin is significantly reduced in vivo [65–67]. Endothelium-dependent hyperpolarization factors (EDHF)-mediated relaxation is also reduced in arteries of women with preeclampsia [68]. Vessels isolated from preeclamptic patients have increased responsiveness to vasoconstrictors including potassium chloride (KCl) and arginine vasopressin (AVP) and have limited vasodilatation in response to acetylcholine in vitro [69]. Studies using animal models of preeclampsia have reported comparable findings [70]. Moreover, vascular dysfunction in preeclampsia can be manifested as augmented arterial stiffness and remodeling [71].

Although there may be multiple factors that contribute to endothelial dysfunction in preeclampsia, poor placentation and ischemic placenta, associated with acute atherosis or thrombosis, are considered critically contribute to endothelial dysfunction [72]. Recent reports have suggested that maternal factors including obesity, hyperlipidemia, insulin resistance, and inflammation-associated coagulation factors alteration are correlated to impaired placentation [43, 73, 74]. These conditions further augment oxidative stress and disrupt both maternal and perinatal endothelial functions that lead to organ hypoperfusion [43]. Strikingly, incubation of resistance vessels from normotensive pregnant women with plasma from either preeclampsia patients or pregnant women who would later develop preeclampsia also results in a reduction of endothelium-dependent relaxation ex vivo, which highlights the contribution of the imbalanced circulating factors in the development of endothelial dysfunction during preeclampsia [75, 76]. These mechanisms lead to endothelial dysfunction that eventually results in hypertension and increased cardiac output, stroke volume, and systemic vascular resistance during preeclampsia, as well as in postpartum, and increase the risk for cardiovascular disease later in life [77, 78]. In addition, growing evidence has linked these placental vascular pathologies with poor fetal growth and adverse birth outcomes [79–81].

**Fig. 2:**
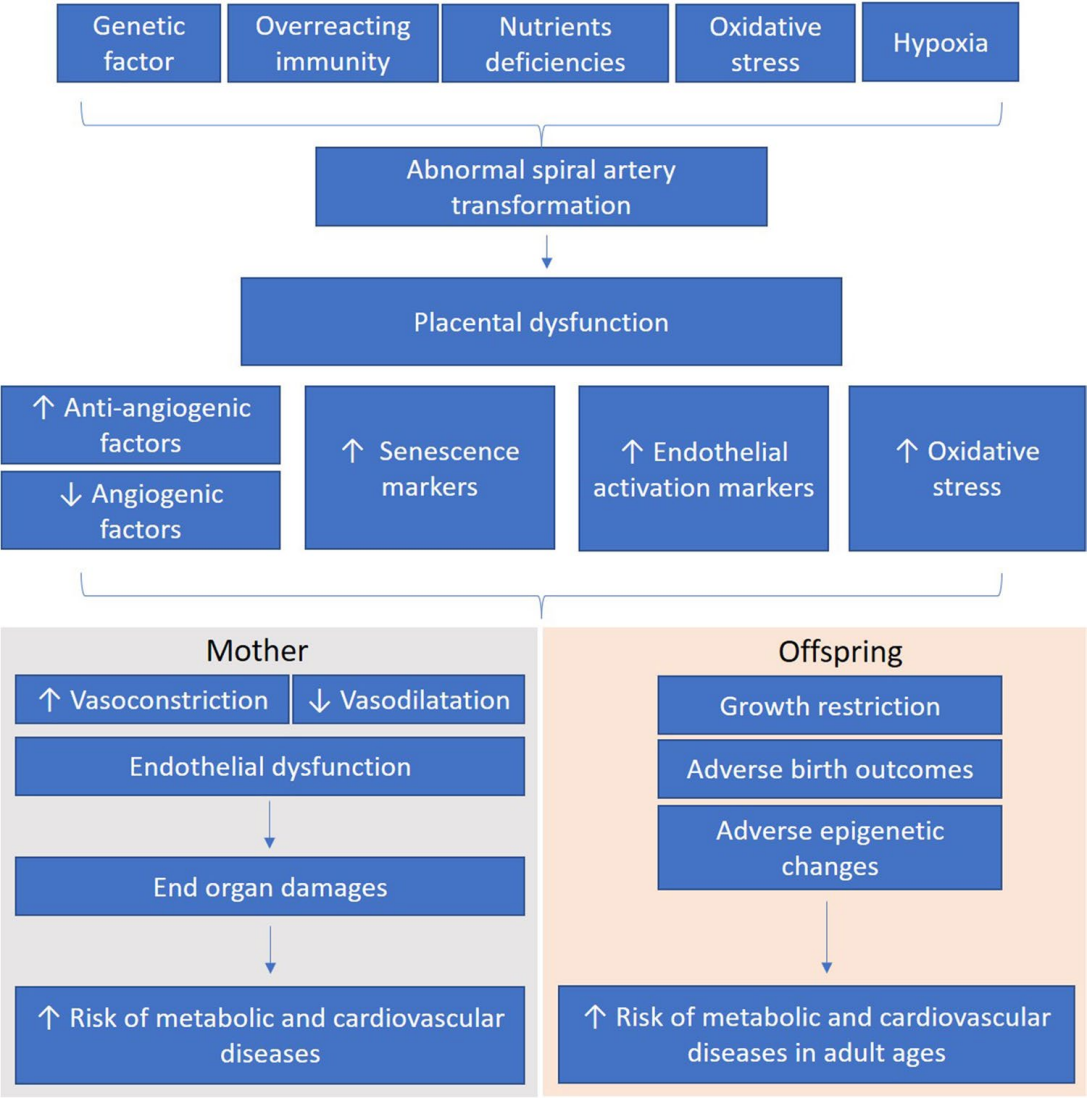
Placental dysfunction in hypertensive disorders of pregnancy. The current understanding of the pathogenesis of placental dysfunction in hypertensive disorders of pregnancy. Placenta plays an important role in the development and severity of preeclampsia. It is proposed that the initiating step in the pathogenesis of preeclampsia is the abnormal placentation that characterized by defective trophoblast cell invasion and uterine vasculature remodeling. Placental dysfunction leads to further imbalance in angiogenic factors, increased placental oxidative stress, placental aging and increased endothelial activation markers in the pathogenesis of preeclampsia. These multiple factors can cause adverse effects in both mother and offspring. Endothelial dysfunction leads to end-organ damages in the mother and causes symptoms of preeclampsia. Placental insufficiency can lead to growth restriction and adverse birth outcomes in the offspring. Preeclampsia can also increase the risk of metabolic and cardiovascular diseases in the mother as well as in the later life of the offspring.

In addition to endothelial dysfunction, premature aging of the placenta has recently been associated with hypertensive disorders of pregnancy and intrauterine growth restriction (IUGR) [82]. Patients with preeclampsia have augmented placental senescence compared to normal pregnant women as evidenced by telomere shortening in trophoblasts [82], while other studies have shown the upregulation of protein and gene expression of senescence-associated secretory phenotype (SASP) components including p16, p21, p53, IL-6, IL-8, plasminogen activator inhibitor-1 (PAI-1), and monocyte chemotactic protein-1 (MCP-1) in the placenta from preeclamptic patients compared to normotensive controls [83–86]. One study has demonstrated that senescence of mesenchymal stem cells, multipotent cells with pro-angiogenic activities, is one of the mechanisms by which angiogenesis is inhibited by systemic inflammation in preeclampsia [87]. The same group has recently revealed the aging phenotype in the adipose tissue and kidney during preeclampsia, suggesting that cellular senescence can be one of the important mechanisms of the pathophysiology of preeclampsia [88]. In addition, immunohistological staining of the preeclamptic placentas revealed the presence of 8-hydroxy-2′-deoxy-guanosine (8-OHdG) [83], suggesting that oxidative stress may cause DNA damage, resulting in the activation of tumor suppressor genes such as p53. In fact, chronic low-grade inflammation can increase placental oxidative stress and endoplasmic reticulum stress in the placenta [89]. Poor placentation is also associated with increased placental oxidative stress and endoplasmic reticulum stress, which can facilitate the pathways of senescence [89]. These lead to apoptosis and cell senescence which result in the loss of proliferative capacity in the placenta (Fig. 2).

### Fetal programming and reprogramming

The sensitivity and susceptibility of the epigenome are high during pregnancy and lactation periods and decrease during life [90]. It is suggested that some adult metabolic and cardiovascular diseases have a fetal origin, which is also referred as fetal programming [91, 92]. Apart from maternal end-organ damages, preeclampsia can leave short-term and long-term adverse effects on the offspring. Indeed, the risk of preeclampsia is increased with family history while a maternal history of preeclampsia increases the risk in her daughter, as well as her son’s female partner [93]. Various reports have suggested the hypothesis that nutrition condition during intrauterine life and perinatal period may have long-lasting effects on the future risk of metabolic and cardiovascular disease through persistence of metabolic and physiological adaptations, which is referred as fetal reprogramming [94–97]. Epigenetic modifications can be induced by diet and drug treatments during pregnancy and lactation periods, even with compounds that are not direct inhibitors or activators of the epigenetic machinery enzymes [98, 99]. These suggest that maternal drug treatment and/or dietary supplementation may be very likely to affect the birth outcome and modulate the future risk of metabolic and cardiovascular diseases in the later life of the offspring by epigenetic mechanisms.

### Endothelial nitric oxide synthase, nitric oxide, and preeclampsia

Endothelial nitric oxide synthase (eNOS) is an important regulator of vascular tone and contributes to the reduction of the uteroplacental resistance during normal pregnancy [100, 101]. eNOS exerts its functions mainly via the production of NO by reduction of L-arginine to L-citrulline [101]. It is suggested that, among different mediators that involved in endothelial dysfunction in preeclampsia, the role of eNOS appears most significant in the development of preeclampsia [102]. NO synthesis can be stimulated by intraluminal flow, shear stress, and vasodilators such as acetylcholine and bradykinin [56, 57]. It has been shown that NO production from the endothelium is increased during normal pregnancy and decreases in preeclampsia [103]. Also, various clinical studies have revealed the association between certain eNOS gene variants (for example G894T, 4b/a, c.894G > T (p.(Glu298Asp)), g.-786 T > C, g.2051G > A, and g.1861G > A) and low serum level of NO in preeclamptic patients [104, 105], while the presence of AGT 704C allele is associated with a reduced risk of developing preeclampsia [105]. Reduced eNOS activity or NO production exacerbates sFlt-1-associated preeclampsia-like phenotype in mice, which is partly through activation of the endothelin system [106].

Indeed, preeclampsia occurs during pregnancy exclusively in humans and certain apes, therefore, researches have been limited since there is currently no animal model which exactly mimics human preeclampsia [107]. Rodents are the most commonly used models for investigating preeclampsia, due to the similarity of cardiovascular adaptations to pregnancy in humans. In general, these rodent models of preeclampsia exhibit an increase in blood pressure (approximately 20–30 mmHg rise in systolic blood pressure), accompanied by reduced renal function [107–109]. NOS inhibition by its nonselective inhibitor L-N^G^-Nitro arginine methyl ester (L-NAME) has been commonly used in rodent models to mimic preeclampsia-like phenotype [110]. Chronic L-NAME treatment in pregnant rat results in hypertension, reduced kidney function and increased placental sFLT-1 expression, while proteinuria is not observed and vascular function is not altered ex vivo [111]. Also, fetal malformations could be a side-effect of L-NAME treatment [112]. sFLT1-infusion in rats is another animal model with preeclampsia-like symptoms and is the only animal model reported to have glomerular endotheliosis [113]. However, the relevance of this animal model requires more detailed studies. The main concern of these treatment-induced model is that the symptoms would be unlikely to subside after delivery of the placenta and is not due to a pregnancy-derived phenotype. On the other hand, Dahl salt-sensitive rats (DSSR) is a genetic model of salt-induced hypertension and has been recently reported to display preeclamptic phenotype (including blood pressure, proteinuria, placental hypoxia and reduced uteroplacental blood flow and fetal growth restriction) even when fed a normal chow diet [114–118]. There are a few helpful reviews that provide detailed discussion on animal models of preeclampsia [107–109, 119].

During normal pregnancy, the production of reactive oxygen species (ROS), such as NO, hydrogen peroxide (H_2_O_2_), hydroxyl radical·OH, superoxide anion O2^−^, and peroxynitrite ONOO^−^ is increased [120]. These physiological ROS are important for the development of placenta and promotion of mitochondrial activity in trophoblasts [121]. In addition, uterine contractions and interventions including diet and exercise may induce mild placental ROS production that is also needed for maintenance of pregnancy and embryo development [120]. It is suggested that eNOS/NO signaling plays an important role in maintaining a healthy pregnancy. However, when ROS production is augmented, eNOS becomes uncoupled [122]. It is also suggested that the uncoupling of eNOS in the hypoxic placenta may further trigger high oxidative stress during preeclampsia [100]. Oxidative stress in the placenta can stimulate the release of apoptotic and necrotic trophoblastic placental debris, anti-angiogenic factors, and proinflammatory cytokines into maternal circulation [123].

On the other hand, sFlt-1 secreted from the placenta can antagonize VEGF signaling, which leads to reduced eNOS activity [61]. Hypertensive disorders of pregnancy are associated with increased levels of factors that inhibit eNOS/NO pathway. Plasma level of asymmetric dimethyl arginine (ADMA), a competitive inhibitor of eNOS, is increased in preeclamptic patients [54], while the expression of a placenta-derived soluble transforming growth factor beta (TGF-β) coreceptor, endoglin, which impairs eNOS activation is also increased [124]. Recent report has also shown that microRNAs (miRs) are upregulated in the human placenta during preeclampsia [125]. These miRs are carried by exosomes and can suppress NO production and eNOS expression by targeting the 3’-untranslated region on eNOS [125].

NO donors have potent vasodilator effect and have been shown to improve blood flow in the fetoplacental circulation during mild preeclampsia [126]. The vasodilator effect of NO signaling is modulated by the interaction between NO and heme-containing proteins, most notably soluble guanylyl cyclase (sGC) [127]. In addition, NO is an endogenous signaling molecule that is involved in the regulation of various important cellular functions. NO can regulate gene expression by either direct interaction with transcription factors or by post-translational modifications of proteins [128]. Recent studies have also revealed the importance of eNOS/NO signaling in modulating the expression of angiogenic factors including PlGF, VEGF, angiopoietins and their receptor soluble angiopoietin-tunica interna endothelial cell kinase 2 (Tie-2), thrombospondin, and anti-angiogenic factors such as sFlt-1 [129, 130]. NO is involved in the transcriptional regulation of histone-modifying enzymes and modulates the activities and cellular localizations of transcription factors through the formation of S-nitrosothiols or iron nitrosyl complexes. [131]. In addition, NO may alter the cellular methylation, acetylation, phosphorylation, ubiquitylation, or sumoylation profiles of proteins and histones [132]. S-nitrosylation is a post-translational modification on the cysteine residue by NO [133]. S-nitrosylated proteins are reported to be involved in different pregnancy-related processes including trophoblast cell migration, immunomodulation, apoptosis, and oxygen delivery [134]. Growing evidence has revealed the association between abnormal placental S-nitrosylation and preeclampsia in both human [135] and rodent [136]. The nitroso-proteomes in the placentas from normotensive and preeclamptic patients are significantly different, while among these downregulated S-nitrosylated-proteins in preeclampsia, annexin A2 is responsible for the activation of angiogenesis [137]. Therefore, reduced eNOS/NO signaling in hypertensive disorders of pregnancy may alter the nitrosylation of proteins which lead to placental dysfunction.

## Dietary supplement

Due to the complex etiology of preeclampsia and safety concerns on drug usage during pregnancy, there is still no effective pharmacological treatments available for preeclampsia yet [3]. An emerging area of interest in this research field is the potential beneficial effects of dietary intervention on reducing the risk of preeclampsia [138]. Recent studies have been focused on the association between deficiencies or excesses of some nutrients and complications during pregnancy, fetal growth and development, and later risk of cardiovascular and metabolic diseases in the offspring.

During pregnancy, nutrient intake during the periods of periconception and pregnancy has significant influences to both the health of mother and the development of the fetus [139]. Pregnancy greatly changes a woman’s metabolism and increases the demands for energy, proteins, vitamins, and minerals. The daily requirement of dietary intake in healthy pregnancy woman is higher than that of a healthy nonpregnant woman [140]. The growth and development of fetus depend completely on the mother; hence, adequate maternal dietary intake is essential for the health of the mother and the development of the fetus. In this sense, pregnancy represents a challenge from a nutritional perspective [141, 142]. Unbalanced diet is a well-known risk factor for cardiovascular diseases and diabetes; hence it may also play a role in the pathophysiology of hypertensive disorders of pregnancy [143]. In addition, imbalanced serum levels of nutrients have been associated with increased inflammation, oxidative stress, and dyslipidemia [144]. Nutrient status including increased serum triglyceride and fatty acids, and reduced levels of magnesium, zinc and vitamins, as well as low calcium intake have been associated with increased risk of pre-eclampsia [145, 146].

An ideal nutrient supplement and/or therapy for pregnancy-related complications should have protective effects in relieving the symptoms of preeclampsia in the mother, improving fetal growth and survival, as well as reducing the disease risk of the offspring at adult ages. Although, the exact effects and mechanisms of nutrition in the alleviating preeclampsia are still debatable, growing number of studies has provided supporting evidence for the potential use of supplements in alleviating preeclampsia. Currently, two main mechanisms have been proposed: (i) Nutritional supplementation shows pharmacological effects in women with dietary intake of nutrients above the recommended daily amount [147]; (ii) nutritional supplementation may achieve benefit via normalizing the deficiency in women with inadequate nutrition [148]. Here, we summarize some of the supplements and their documented beneficial effects in hypertensive disorders of pregnancy.

### L-arginine

As mentioned above, NO is a potent endothelium-derived vasodilator and defective eNOS/NO signaling has been documented in preeclampsia. NO is produced by eNOS which uses L-arginine as substrate. Therefore, the bio-availability of L-arginine is important to maintain the endothelial adaptive regulatory mechanisms for vasodilatation in healthy pregnancy. Arginine is a semi-essential amino acid and the precursor of various biological pathways including the urea cycle and the production of NO and polyamines [149, 150]. Arginine may regulate many metabolic pathways that are crucial to reproduction, growth, and health [151], while the role and functions of NO have been discussed above. It is well documented that the administration of L-arginine improves vascular function in atherosclerosis and peripheral vascular diseases [152–154].

Recently, it has been shown in some animal and clinical studies that L-arginine have the potential to alleviate preeclampsia [155–157]. In a recent systematic review and meta-analysis of randomized controlled trials, L-arginine supplementation has been shown to increase plasma NO concentration in IUGR pregnancies and decrease the risk of preeclampsia [158]. In addition, L-arginine supplementation can improve birth weights of offspring in both hypertensive and IUGR pregnant women [158]. Moreover, it has been suggested that intravenous infusion of L-arginine, but not oral administration, significantly increases NO concentrations and birth weights in IUGR pregnancies [158]. L-arginine has also been shown to alleviate the pathogenesis of malaria-induced adverse birth outcomes in pregnancy [159, 160].

Apart from NO, several metabolites of arginine, such as polyamines, are important nutrients required in multiple stages of pregnancy, including implantation, early embryogenesis, fetal growth, and placental development [161]. Polyamines, such as putrescine, spermine, and spermidine, will interact with negatively charged molecules, including DNA, RNA, acidic proteins, and phospholipids [162, 163]. Indeed, polyamine metabolism plays an important role in placental function during pregnancy [164]. Reductions in polyamine bioavailability in pregnant rodent models have been associated with abnormal placentation and fetal growth restriction [165, 166]. Polyamines are involved in the regulation of the inflammatory response due to their antioxidant properties [167] and modulate T cell responses [168]. Treatment with spermine inhibits lipopolysaccharide (LPS)-mediated production of pro-inflammatory cytokines, such as TNF-α and IL-6 in mouse macrophages [169]. The beneficial effects of polyamines may be attributed to the regulation of pathways involved in endothelial cell migration, proliferation, protein synthesis, and pro-angiogenic gene expression [164]. However, the underlying molecular pathways by which polyamines are involved in angiogenesis remain unclear. Recent study also reveals the epigenetic effects of placental polyamines via regulating acetyl-CoA level and histone acetylation [161].

These observations raise the possibility of practical dietary L-arginine supplementation during pregnancy as a NO donor to reduce the risk of preeclampsia. Nevertheless, although L-arginine is an inexpensive supplementation with a known safety profile in pregnancy, the bioavailability of arginine is relatively low, as 40% of oral arginine is catabolized by the intestine and another 9% is metabolized by the liver [159]. Therefore, the challenge of considering L-arginine as a dietary supplement targeting preeclampsia would be the low bioavailability.

### L-citrulline

L-citrulline is an amino acid that is also the natural precursor and metabolite of L-arginine. L-citrulline is converted to L-arginine by arginosuccinate synthase. Activation of arginosuccinate synthase in cells isolated from preeclamptic patients has been shown to improve endothelial function, associated with increased NO production and reduced oxidative stress [170, 171]. On the other hand, compared to arginine, citrulline has been shown more effective to promote NO production as it can bypass hepatic metabolism, and not metabolized by arginase [159]. A few evidence has suggested that the protective effect of citrulline may not be analogous with arginine [172], and these two amino acids regulate gene expression in different manners [173]. Citrulline has also been shown to improve protein anabolism, increase nitrogen balance in rats [174], and enhance muscle protein synthesis in human [175] more efficiently than arginine. In addition, the usage of citrulline has an even greater safety profile than that of arginine, as none of the clinical trials have reported any adverse effects [176]. Therefore, these raise the hypothesis that citrulline can be a better supplementation than arginine in targeting hypertensive disorders of pregnancy.

In our recently published study, we examined the effect of citrulline supplementation in a rat model of preeclampsia [177]. We have demonstrated the protective effects of citrulline supplementation in the Dahl salt-sensitive rats (DSSR). Citrulline supplementation in DSSR leads to the reduction of maternal blood pressure and markers of preeclampsia (sFlt-1). Pup-to-placenta weight ratio and maternal vascular function are improved by citrulline supplementation. In addition, the beneficial effects of citrulline in ameliorating placental fibrosis and senescence, and promoting angiogenesis in the placentas have been observed, in parts, attributable to the downregulation of toll-like receptor 4 (TLR4) and nuclear factor κB (NF-κB) in the placenta [177]. Coherently, another study has demonstrated the effect of L-citrulline supplementation in a mouse model of preeclampsia [178]. This study shows that L-citrulline supplementation can reduce blood pressure, increase vascular glycocalyx volume, and improve maternal vascular function ex vivo at gestation day 17.5 in the preeclampsia-like mouse model [178]. The beneficial effects of citrulline in maternal vascular function appear, in part, attributable to eNOS/NO signaling. In addition, L-citrulline supplementation has also been shown to enhance fetal growth and protein synthesis in rats with intrauterine growth restriction [179]. Maternal L-citrulline supplementation can prevent 50% caloric restriction-induced low nephron number and renal dysfunction in the offspring, although increased blood pressure has been observed in the offspring [180]. The effects in developmental programming of kidney disease and hypertension are associated with reduced ADMA in the plasma [180]. Also, maternal supplementation with citrulline has been shown to have long-term antihypertensive effects in the offspring of spontaneous hypertensive rats [181]. Moreover, maternal L-citrulline treatment prevents prenatal dexamethasone-induced programmed hypertension by restoration ADMA/NO balance, alterations of renin–angiotensin system and sodium transporters, and epigenetic regulation by histone deacetylases in mice [182].

## Micronutrients

Micronutrients, including fatty acids, vitamins, and minerals, are chemical substances that are required in small amount but are important in regulating the metabolic and biochemical processes of the body [183]. Deficits in any of them can lead to deficiencies in growth and development, as well as abnormal physiological functions, and immunity. Current evidence supports the idea that deficiencies of these micronutrients have adverse effects in maternal health and the outcome of pregnancy.

### Fatty acids

Lipid and fatty acids are involved in the generations of free radicals [184]. High-fat diet is atherogenic, while polyunsaturated fats are substrate for lipid peroxidation and have been reported to be increased in the diet of preeclamptic women [185]. On the other hand, omega-3 long-chain polyunsaturated fatty acids (n-3 PUFAs) have been suggested to be involved in the prevention of preeclampsia. Studies have revealed that levels of n-3 PUFAs were lower in erythrocytes of women with preeclampsia [186]. n-3 PUFAs are essential for fetal tissue formation [187]. In addition, n-3 PUFAs have been shown to have protective effects in cardiovascular function and alleviating inflammation [188, 189]. Eicosapentaenoic acid (EPA, 20:5), and docosahexaenoic acid (DHA, 22:6) have been associated with beneficial effects in vasodilation [190]. In addition, n-3 PUFAs have been shown to increase eNOS activity and NO production [191, 192]. The beneficial effects of n-3 PUFAs are suggested by the evidence that there is a reduced rate of preeclampsia in population that have large quantities of fish or fish oil intake [193]. In a recent systematic review and meta-analysis, the comparison of clinical trials has also suggested that n-3 PUFAs supplementation played a protective role against the risk of preeclampsia in women with low-risk pregnancies [194], probably by reducing placental inflammation and oxidative stress [195]. In a recent umbrella review of meta-analyses of randomized trials, n-3 PUFAs supplementation during pregnancy can exert beneficial effects in improving birth weight, preterm delivery, and post-partum depression, and reducing cardiometabolic risk factors in pregnant mothers, as well as can improve anthropometric measures, immune system, and visual activity in infants [196]. It has also been recently reported the effect of maternal intake of n-3 PUFAs during pregnancy influences the offspring DNA methylation [197]. Also, it has been recently shown that maternal n-3 PUFAs supplementations are closely correlated to infant telomere length [198]. However, the long-term effect of maternal intake of n-3 PUFAs in reducing future risk of metabolic and cardiovascular diseases in the offspring is unknown.

### Calcium

Calcium supplementation has been reported to reduce the risk of preeclampsia [199]. Indeed, calcium is the most-studied micronutrient in relationship to preeclampsia. Various epidemiological studies have demonstrated the association between reduced calcium intake and preeclampsia [200, 201] and women with low calcium intake (< 800 mg/day) are considered at increased risk of preeclampsia [21]. Several randomized controlled trials have been performed to investigate the effect of calcium supplementation. The analysis of these trails has indicated a 32% reduction of the incidence of preeclampsia with calcium supplementation [199], while this beneficial effect is most prominent in low baseline calcium intake groups. However, one of the largest trials to date reported no effect of calcium supplementation on preeclampsia [202], probably due to the inclusion of pregnant women with adequate dietary calcium intake. Therefore, current evidence has supported the hypothesis that the incidence of preeclampsia can be reduced by calcium supplementation, while women with low calcium intake seem to be more likely to benefit from calcium supplementation [203]. In addition, prenatal supplementation with high-dose calcium has been shown to reduce the prevalence of gestational hypertension, serious maternal morbidity or death, and preterm birth [199]. Moreover, maternal calcium supplementation has been shown to improve the postpartum maternal bone health [204, 205], as well as to reduce the risk of increased systolic pressure in the offspring [206].

### Vitamins

A recent systematic review and meta-analysis have shown the positive correlation between lower rate of preeclampsia and calcium and vitamin D intake [207]. Vitamin D is a pre-hormone, that can also be endogenously produced by the skin when exposed to UV-B. Vitamin D plays an important role in preventing bone diseases and improving calcium metabolism [208]. Recently, vitamin D has also been shown to regulate the expression of signature developmental and angiogenic genes in the placenta [209–211]. Vitamin D deficiency before 22 weeks of pregnancy is a strong and independent risk factor for preeclampsia [208]. Maternal vitamin D insufficiency could also lead to preterm birth, small for gestational age or IUGR and gestational diabetes mellitus [212]. The active vitamin D metabolite, 1,25-dihydroxyvitamin D3 [1,25-(OH)2D3], exerts immunosuppressive activity. It has anti-proliferative effect on Th1 cells and inhibits the secretion Th1 cytokines [213]. Deficiency of vitamin D is significantly associated with placenta calcification and aging [214]. Various studies have provided evidence supporting that vitamin D supplementation can reduce the incidences of preeclampsia [215–217]. Some recent studies have shown that vitamin D supplementation can improve mitochondrial function, reduce inflammation in the placenta and preserve placental functions [218, 219]. A few trails have shown that vitamin D supplementation can reduce the risk of having a preterm birth [220, 221], as well as improve fetal growth and peripheral blood flow in the fetus [222]. One study has shown that low vitamin D status is associated with lower adiposity at birth, but a greater offspring adiposity at age of 6 years [223]. However, the long-term effect of maternal vitamin D supplementation in the offspring is still unclear.

Vitamins C and E are important non-enzymes that involve in the endogenous cellular antioxidant system. Vitamin C is water soluble while vitamin E is lipid soluble [224]. Vitamin C level is decreased in women with preeclampsia [225, 226] while vitamin E has been shown to be reduced in some [225, 226] but not all cases [227, 228]. Vitamin E is consistently reduced in severe cases of preeclampsia [229, 230]. Adequate dietary intake of vitamins C and E appears to be mandatory to prevent oxidative stress [231]. Indeed, antioxidants have been proposed as prophylactic agents for preeclampsia [232, 233]. Various studies have demonstrated the beneficial effect of vitamin C and E supplementation in reducing preeclampsia by 4–12% depending on the risk level of the pregnant woman [234, 235]. Vitamin C or E supplementation can reduce the systolic blood pressure in the preeclampsia patient [235]. Supplementation with vitamins C and E can has been shown to reduce PAI-1/PAI-2 ratio during gestation [233], suggesting the beneficial effects of vitamins C and E may be attributed to alleviating endothelial senescence and placental insufficiency. Vitamin C administration improves endothelial function, as evidenced by the increased flow-mediated dilatation measured by in vivo ultrasonography, in previously preeclamptic women [55]. However, some of the studies did not observe any significant effects of vitamins C and E [147, 236]. Nevertheless, a recent meta-analysis has indicated that multivitamins supplementation can significantly reduce the risk of preeclampsia [237], suggesting that the beneficial effects of vitamins may be interrelated.

## Resveratrol

Resveratrol (3,5,4′-trihydroxy-trans-stilbene) is a polyphenol found in grape fruits and can be obtained from drinking red wine [238]. Resveratrol has been widely studied on its anti-oxidant and anti-inflammatory activities [239–241]. The protective effects of resveratrol have been demonstrated in cancer, cardiovascular, metabolic, and neurodegenerative diseases [242–244]. Resveratrol exerts its beneficial effect in cardiovascular protection by increasing the production of NO, partly by, upregulating eNOS expression, stimulating eNOS activity, and preventing eNOS uncoupling [245]. Also, resveratrol can modulate the function of immune cell and inhibit immune cell infiltration [246, 247].

Resveratrol supplementation improves the efficacy of oral nifedipine treatment in preeclampsia [248]. Maternal resveratrol consumption could decrease inflammation and oxidative stress in placental and embryonic tissues [249]. Also, resveratrol treatment has been shown to increase uterine artery blood flow and fetal oxygenation, upregulate antioxidant enzymes in the placenta, reduce markers of endothelial dysfunction, and enhance placental and fetal weight in a rat model of severe hypoxia [250]. The expressions of endothelial dysfunction markers, including ICAM-1, von Willebrand factor, and Caspase-3, in endothelial cells and umbilical arteries from preeclampsia patients are coherently attenuated by resveratrol treatment [251]. A recent study has revealed that resveratrol ameliorates preeclampsia by upregulating VEGF through miR-363-3p-mediated pigment epithelium-derived factor (PEDF) downregulation [252]. Moreover, a systematic review of 31 studies has suggested that resveratrol possesses epigenetic effects in the development of placenta and fetal tissues during the gestational period [253]. Various animal studies have demonstrated the fetal reprogramming effects of maternal resveratrol supplementation that attenuate obesity, prevent hepatic steatosis, and improve insulin sensitivity and islet dysfunction in the offspring [254, 255].

Recently, the effects of sirtuin 1 (SIRT1) on the biological functions of trophoblasts and endothelial cells have gradually emerged, and the serum and placental level of SIRT1 is reduced in preeclampsia [256–258]. SIRT1, which can be activated directly or indirectly by resveratrol, is also known as a longevity enzyme. SIRT1 activity is controlled by intercellular nicotinamide adenosine dinucleotide (NAD) levels and is involved in transcriptional regulation of a large number of genes that are involved in different cellular functions [238]. SIRT1 can also activate eNOS and enhance endothelial function [238] and is an important player in regulating vascular function and preventing vascular aging [259]. The beneficial effects of resveratrol in cardiovascular diseases are comparable to calorie restriction or SIRT1-overexpression models in vivo [260, 261]. A recent study has demonstrated that treatment of recombinant SIRT1 protein can decrease maternal blood pressure and improves angiogenic imbalance, inflammation, and pregnancy outcome in a rat model of preeclampsia [257]. High levels of sFlt-1, TNF-α, and IL-6 in maternal plasma are normalized by the treatment of recombinant SIRT1 protein [257]. In another recent study, downregulation of SIRT1 has been correlated to the accelerated senescence of syncytiotrophoblast via downstream targets contributing to the regulation of the cell cycle, extracellular matrix production, and cytoskeleton reorganization, which lead to premature placental aging in preeclampsia [258]. Resveratrol-induced SIRT1 activation abrogates senescence in trophoblast-derived BeWo cells in vitro [258], suggesting the beneficial effect of resveratrol in ameliorating placental senescence in preeclampsia. In addition, resveratrol has also been studied for the beneficial effect on modulating gut microbiota [98, 245, 262]. Recent studies have discussed the causal association between gut dysbiosis and preeclampsia [263–265]. These raise the possibility that beneficial effect of resveratrol in alleviating preeclampsia is partly via gut microbiota remodeling.

## Conclusion and future directions

Pregnancy-associated hypertensive disorders represent an important cause of maternal and infant mortality and morbidity, as well as increasing the risk for future metabolic and cardiovascular disease for both mother and offspring. Although the knowledge of pregnancy-associated hypertensive disorders has increased dramatically over the past years, the role of diet and the potential use of dietary supplements or therapy targeting preeclampsia has not been adequately studied. Currently, different clinical trials and meta-analyses have been performed to evaluate the effect of different dietary supplements on the development of preeclampsia (Table 1). However, these studies are mostly limited by the fact that the pathogenesis of preeclampsia could be multifactorial, severity of preeclampsia can be varying and heterogeneity between studies is very high. In addition, in most clinical cases of preeclampsia, it is not possible to discriminate the cause and effect between nutrient deficiencies and preeclampsia. Therefore, there is a need to apply novel animal models of preeclampsia for future studies in this field. To improve study quality, we must also try to explore the effects of these interventions and/or therapeutics in three important aspects: the maternal syndrome, fetal growth and survival, and the disease risk of the offspring at adult ages. Nevertheless, this review has summarized the current understanding of the pathophysiology of pregnancy-associated hypertensive disorders in a vascular perspective. We have also listed out the beneficial effects of a few dietary supplements targeting preeclampsia. This review aims to provide some potential insight and brainstorming for the future direction of studies with dietary interventions in preventing preeclampsia.

**Table 1:**
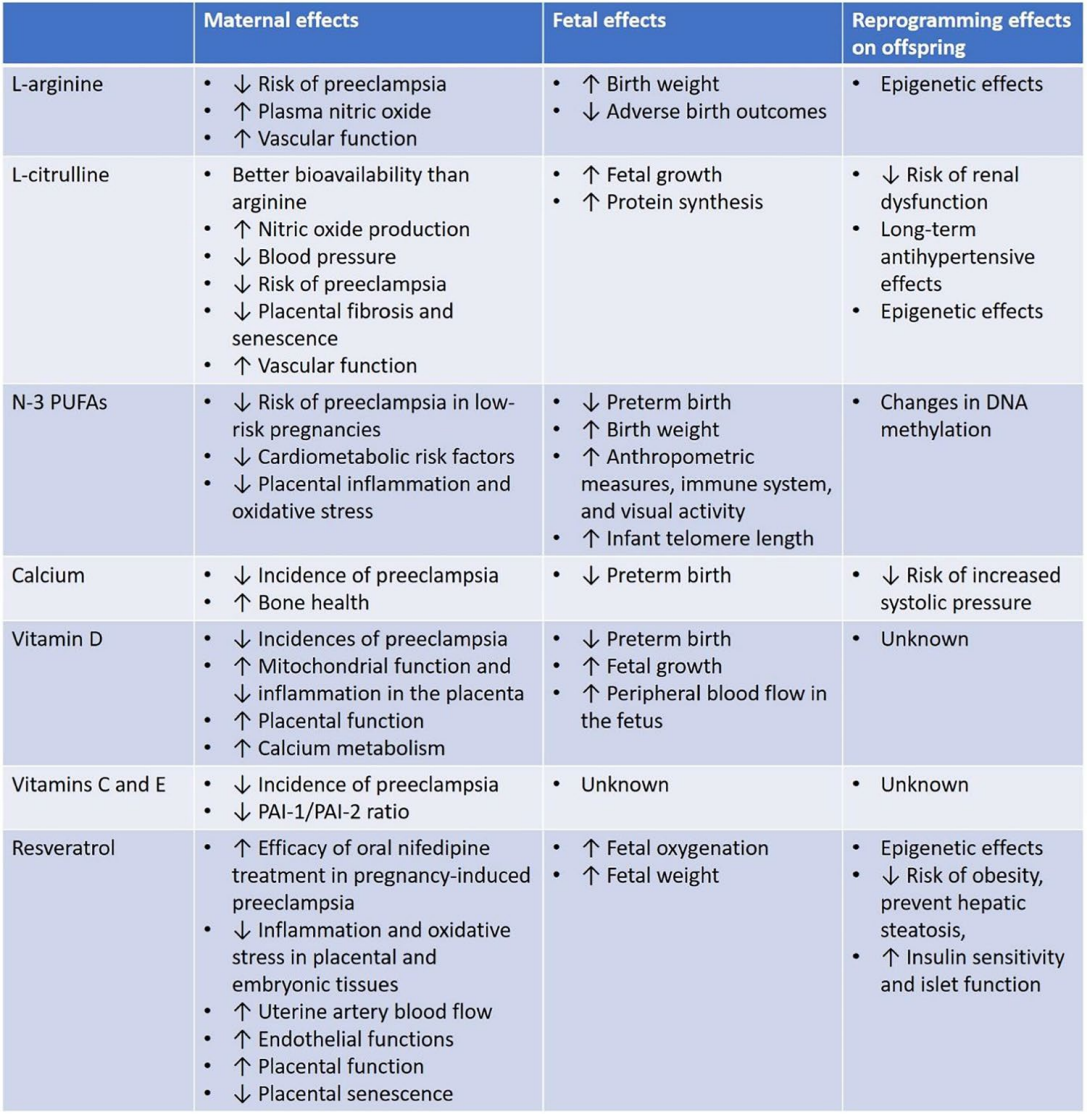
Summary of the potential beneficial effects of various supplements in hypertensive disorders of pregnancy reported in clinical and animal studies, focusing on the three aspects: maternal effects, fetal effects, and effects in risk of future diseases in the offsping

## Data Availability

Not applicable.
